# Supplementary material for: Three-Source Partitioning of Methane Emissions from Paddy Soil: Linkage to Methanogenic Community Structure
Source: Int J Mol Sci. 2019 Mar 29;20(7):1586. doi: 10.3390/ijms20071586 (PMC6479939; doi:10.3390/ijms20071586)
Supplement: Supplementary file 1 [file ijms-20-01586-s001.pdf]

Supplementary Figures

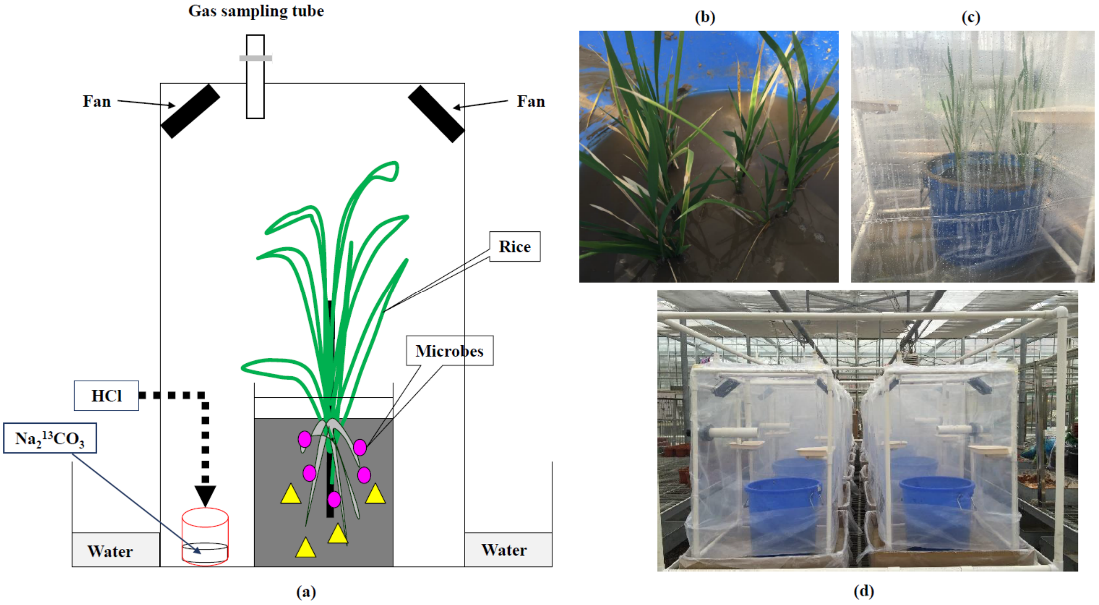

Fig. S1 Experimental system. The chamber was set inside a sealed plastic chamber with transparent and sealed film outside. It had 490 L (70 cm × 70 cm × 100 cm), and the bottom was filled with water to prevent the leaching of the gases. The soil container was 30 cm wide (diameter) and 30 cm high. The soil inside was 20 cm high, packed two weeks before transplanting. It was 20 kg per column-shaped container. The irrigation was made after base fertilizer and maintained at 5 cm above the soil surface.

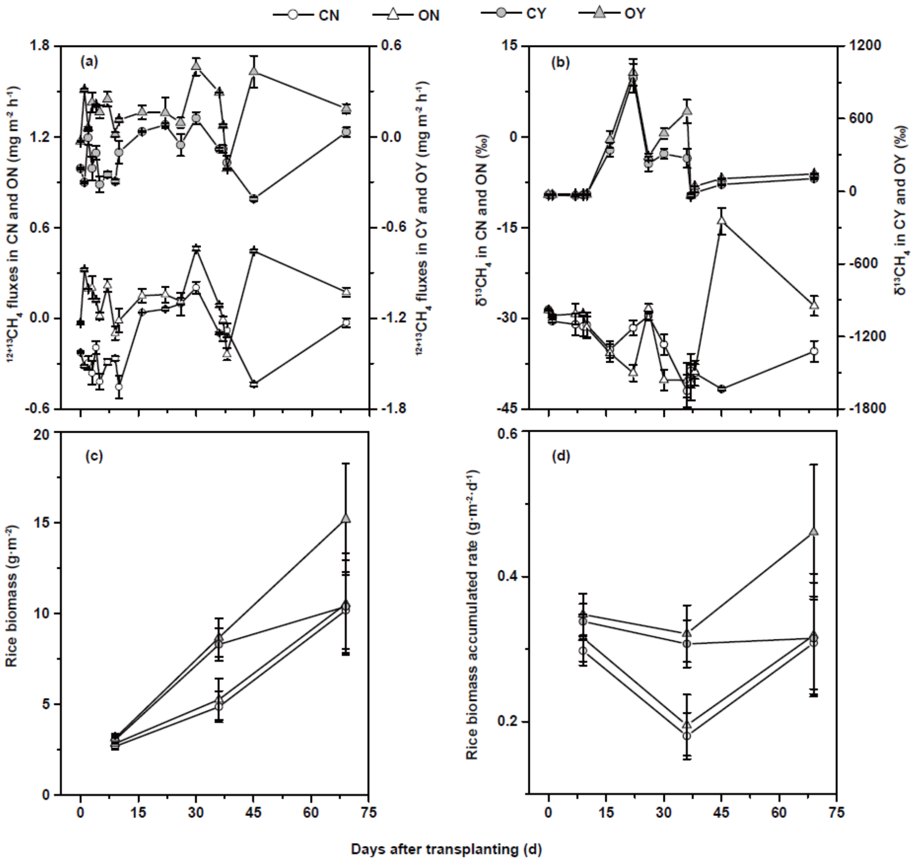

SFig. S2 $^{12+13}CH_4$ fluxes, $\delta^{13}CH_4$ and rice biomass. In Supplementary Figure 2a, the $^{12+13}CH_4$ fluxes reached the maximum at the 30th day after transplanting, wherein CN, ON, CY and OY were 0.20, 0.46, 0.12 and 0.46 mg·m^{-2}·h^{-1}, respectively. Overall, the average of $^{12+13}CH_4$ fluxes in organic fertilizer treatment was 0.15 mg·m^{-2}·h^{-1}, significantly higher than that of no fertilizer treatment (-0.15 mg·m^{-2}·h^{-1}). In Supplementary Figure 2b, $\delta^{13}CH_4$ in CY and OY reached their maximum, and with 935.99‰ and 981.11 ‰ on the 22nd after rice transplanting. According to equation (5), the final $\delta^{13}CH_4$ in organic fertilizer treatment was 171.19 ‰, which was 21.37% higher than that of no fertilizer treatment (141.05 ‰). Besides, the rice biomass accumulated from 2.92 g·m^{-2} to 11.58 g·m^{-2} were shown in Supplementary Figure 2c. As for the accumulation rate of rice biomass (Supplementary Figure 2d), it was firstly decreased at the tillering stage, and then increased at the booting stage. The average accumulation rates for organic fertilizer and no fertilizer treatment were 0.33 and 0.29 g·m^{-2}·d^{-1}, respectively.

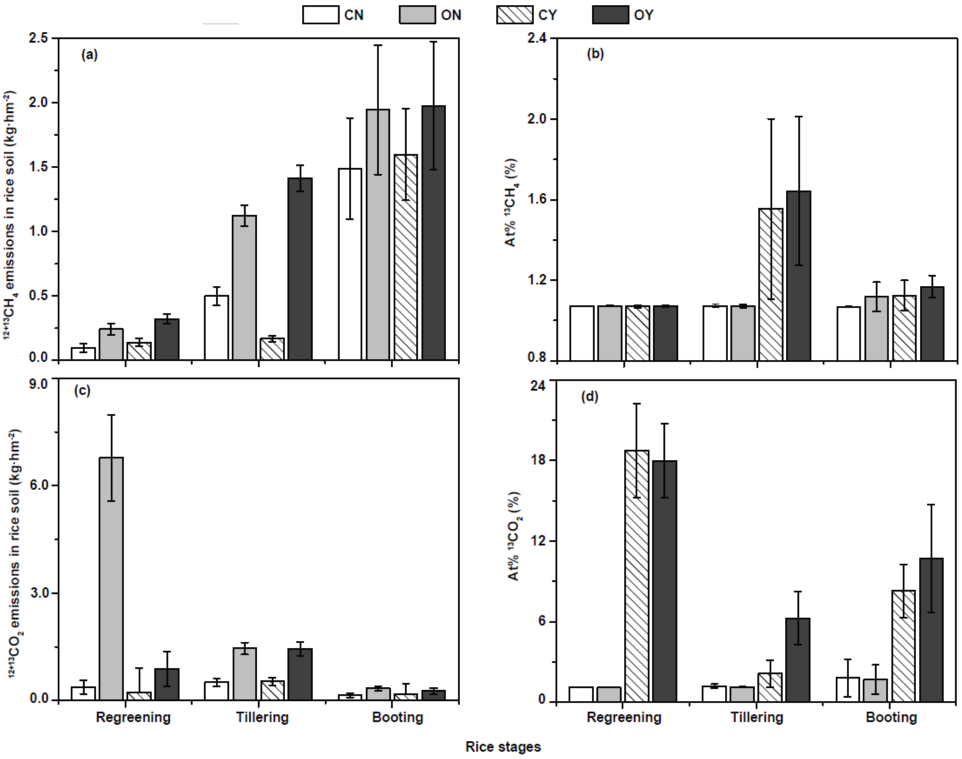

SFig. S3 $^{12+13}C$ emissions and atom percentage. In Supplementary Figure 3a, the total $^{12+13}CH_4$ emissions in organic fertilizer treatment were averaged 3.51 kg·hm^{-2}. It increased 75.50% compared to the no fertilizer treatment (2.00 kg·hm^{-2}). The atom percentage of $^{13}CH_4$ (at% $^{13}CH_4$, Supplementary Figure 3b) in no ^{13}C labelling treatments was 1.07%. However, it varied from rice growth stages for ^{13}C labelling treatments: 1) at% $^{13}CH_4$ in CY and OY were both 1.07% at the regreening stage; 2) at% $^{13}CH_4$ in CY and OY maximized with 1.55% and 1.64% at the tillering stage; 3) and at% $^{13}CH_4$ in CY and OY were 1.12% and 1.17% at the booting stage. In Supplementary Figure 3c, the total $^{12+13}CO_2$ emissions from rice soil in CN, ON, CY and OY were 1.05, 8.57, 0.96 and 2.60 kg·hm^{-2}, respectively. As for the atom percentage of $^{13}CO_2$ (at% $^{13}CO_2$, Supplementary Figure 3d), the average at% $^{13}CO_2$ in no ^{13}C labelling treatments was 1.33%. At the regreening stage, at% $^{13}CO_2$ in CY (18.75%) and OY (17.97%) were higher than other stages.

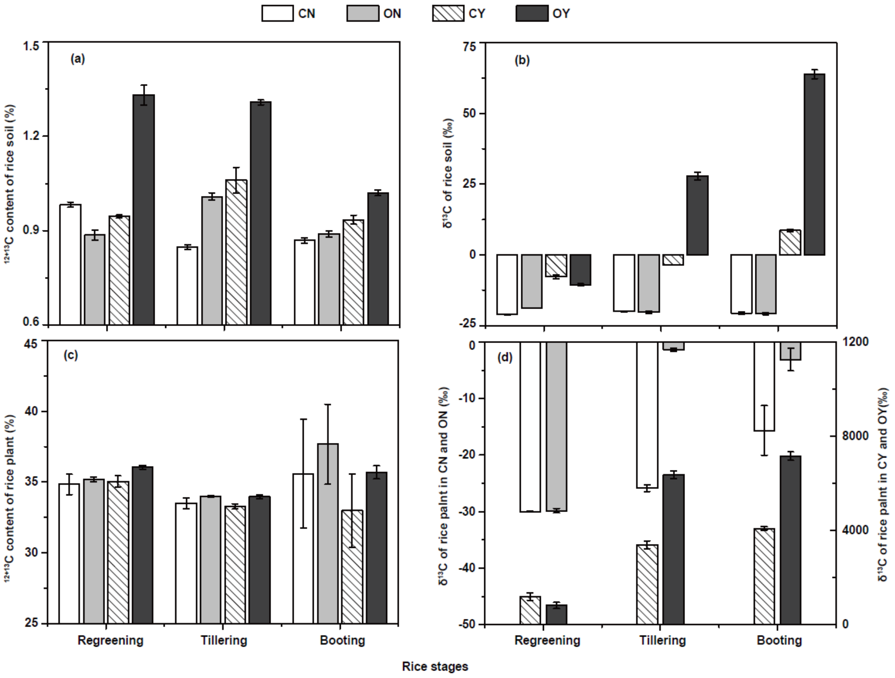

SFig. S4 Soil and rice plant C. In Supplementary Figure 4a, the average soil $^{12+13}C$ in organic treatment (1.07%) was higher than that in no fertilizer treatment (0.94%). In Supplementary Figure4b, the soil $\delta^{13}C$ with no ^{13}C labelling were average -20.31‰. However, the soil $\delta^{13}C$ of CY and OY were increased from regreening stage to booting stage. At the booting stage, the soil $\delta^{13}C$ were 8.60‰ in CY and 63.88‰ in OY. In Supplementary Figure 4c, the rice plant $^{12+13}C$ was 34.81% on average. In Supplementary Figure 4d, the rice plant $\delta^{13}C$ for no ^{13}C labelling was -29.91‰, but it was 1.00% for ^{13}C labelling at the regreening stage. After that, the rice plant $\delta^{13}C$ of CY and OY continued increasing2. Results.
